# A journey in bioinspired supramolecular chemistry: from molecular tweezers to small molecules that target myotonic dystrophy

**DOI:** 10.3762/bjoc.12.14

**Published:** 2016-01-25

**Authors:** Steven C Zimmerman

**Affiliations:** 1 Department of Chemistry, University of Illinois at Urbana-Champaign, Urbana, Illinois 61801, United States

**Keywords:** catenanes, intercalation, macrocycles, multi-target drug discovery, RNA recognition, RNase mimic

## Abstract

This review summarizes part of the author’s research in the area of supramolecular chemistry, beginning with his early life influences and early career efforts in molecular recognition, especially molecular tweezers. Although designed to complex DNA, these hosts proved more applicable to the field of host–guest chemistry. This early experience and interest in intercalation ultimately led to the current efforts to develop small molecule therapeutic agents for myotonic dystrophy using a rational design approach that heavily relies on principles of supramolecular chemistry. How this work was influenced by that of others in the field and the evolution of each area of research is highlighted with selected examples.

## Review

### Early childhood and overview

**Figure 1:**
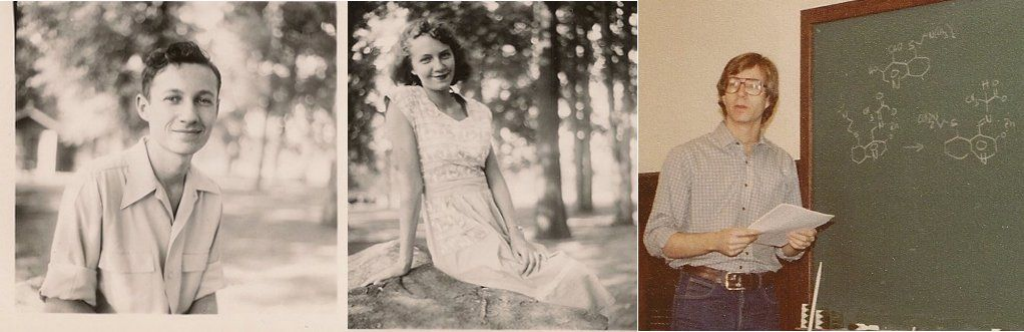
Photographs of Howard E. Zimmerman (July 5, 1926–February 12, 2012) (left) and Jane Zimmerman (née Kirschenheiter) (December 24, 1928–January 21, 1975) (center) as young adults, and the author (right) giving a Breslow group meeting presentation in graduate school (Havemeyer Hall, Columbia University).

I was born on October 8, 1957 in Evanston, Illinois, the second of three boys. Our parents, Howard E. Zimmerman and Jane Zimmerman, née Kirschenheiter, were very much in love and also remarkably different people. My mother was a rebellious, direct-speaking, very liberal, yet religious Christian who never graduated from high school. My father was a soft spoken, politically conservative, nonpracticing Jew, who not only obtained a B.S. and Ph.D. from Yale University, but went on to do postdoctoral work with Robert Burns (R.B.) Woodward at Harvard University. He was the first on either side of the family to get a college degree. With the exception of my father, my family was primarily working class (mailman, construction worker, Navy man, etc.). Family photographs are shown in Figure 1.

The clash of cultures in the family challenged my sense of identity and I grew up feeling like a foreigner, an outsider, in a homogeneous, white, Christian, middle class-neighborhood. My remarkable mother made sure we appreciated our father’s heritage. Although we were raised with traditional Christian holidays, in early adulthood I recognized in myself a sense of humor and an outlook on life that was distinctly Jewish. I was drawn to the books of Saul Bellow and Isaac Bashevis Singer, and New York City. What does any of this have to do with chemistry? Chemistry provided a sense of belonging and identity from an early age simply because my father was immersed in an occupation I didn't understand but recognized as being very exciting. His passion for his work was obvious, so as a young boy I told people I also wanted to be a chemist. Furthermore, the long line of remarkably talented chemistry graduate and postdoctoral students that came to my house for Z-group parties were like an extended family. They were all “cool” people and some, for example, John McCall and Laren Tolbert even served as babysitters. This was a family I wanted to join. The occasional visiting faculty member solidified my choice of chemistry as a career. What child wouldn’t be excited by Koji Nakanishi cutting ropes in two only to have them magically reconnect!

### Undergraduate and graduate studies and an NSF-NATO postdoc

The Department of Chemistry at the University of Wisconsin was an extraordinarily stimulating place in the mid to late 1970s. I was fortunate to do undergraduate research with Professor Hans J. Reich, investigating the mechanism of the singlet oxygen reaction with alkenes and studying the oxidation of selenide/sulfide mixtures using ozone and singlet oxygen [1]. In my senior year, I took three graduate level courses, which was an amazing experience. Professors Charles P. (Chuck) Casey and Harlan L. Goering taught the physical organic course (Chem 641), Professor Barry M. Trost and Edwin Vedejs taught the synthesis course (Chem 841) and Hans Reich a more informal, once-a-week, mechanisms (arrow-pushing) class.

For each lecture, Trost or Vedejs passed out several pages describing various methods of synthesizing several natural product substructures with a rather lengthy bibliography. I naively thought that this bibliography was an assigned reading list rather than a list for future reference. It was a wonderful mistake that led to my learning an enormous amount of exciting synthetic chemistry. Indeed, in going to Columbia University for graduate school I had every intention of working for Professor Gilbert Stork or W. Clark Still. But Professor Ronald Breslow’s enzyme reaction mechanisms course, my first class ever on anything resembling biology or biochemistry, was so exciting that I decided to join the Breslow group and work on pyridoxal/pyridoxamine enzyme analogs [2–4]. Not only was Ron Breslow a wonderful and inspirational mentor, he had built an extraordinarily stimulating group of coworkers that he himself described as “people that you will hear from in the future, not people who will disappear into the woodwork.” Indeed, my labmates during the period from 1979 to 1983 included Jik Chin, Robert Corcoran, Tony Czarnik, Sam Gellman, Don Hilvert, Uday Maitra, Dave Okrongley, Russ Petter, Darryl Rideout (coincidentally a Madison West High School classmate), Alanna Schepartz, Alan Schwabacher, George Trainor, Craig Wilcox, and Jeff Winkler.

Ron Breslow had broad interests, with projects ranging from developing artificial enzymes, to novel anti-aromatic compounds, to remote C–H activation of steroids, to determining hydrocarbon p*K*_a_ values using electrochemistry. The lesson learned, and one I tried to put into practice in my independent career (see below), is that it is very much possible to run a research group focused in quite different areas of chemistry. With an NSF-NATO postdoctoral fellowship, I spent just under two years with Sir Alan Battersby at the University of Cambridge where we completed the total synthesis of sirohydrochlorin, an intermediate in the biosynthesis of vitamin B12. Then in July 1985, it was off to the University of Illinois at Urbana-Champaign.

### Molecular tweezers and a paradigm shift in host–guest chemistry

Developing molecular tweezers was one of the main projects I started in my independent academic career at Illinois. The idea originated at Columbia when I began to teach myself the biochemistry and biology lacking in any of my formal coursework. For example, one summer that process involved taking J. D. Watson’s “Molecular Biology of the Gene” [5] on the subway to a Long Island beach on weekends. The beautiful structure of DNA and its intercalation complexes of aromatic dyes were especially intriguing. In broader reading, I sought to understand better the so-called nearest-neighbor exclusion principle (NEP), wherein intercalators at full saturation of a DNA helix bind every other site (i.e., one intercalator per two base-pairs) [6].

**Figure 2:**
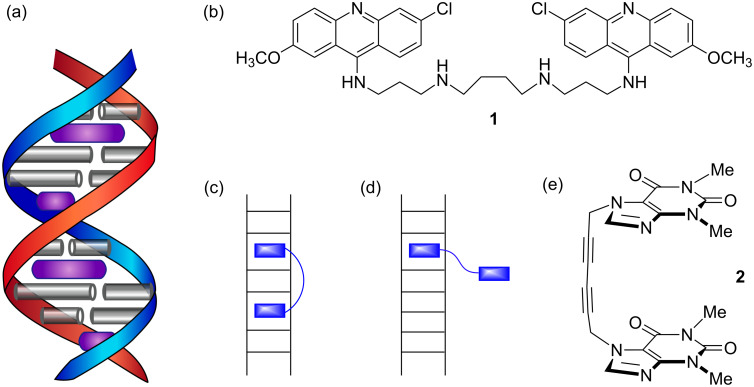
(a) Schematic double helix fully saturated with intercalator (in purple) according to the neighbor exclusion principle (NEP). (b) Bisintercalator with spermine linker. (c,d) Bisintercalator with long linker spanning two base-pairs and short linker preferring mono-intercalation to obey NEP. (e) Whitlock’s “rigid” molecular tweezer.

Figure 2a schematically shows how insertion of monointercalators at full saturation leads to a DNA helix with intercalation sites only half occupied. Le Pecq and coworkers studied bisacridines such as **1** (Figure 2b) [7]. Consistent with the NEP, **1** formed a very tight bisintercalation complex with its spermine-derived linker chain spanning two base-pairs (Figure 2c). However, with shorter linkers that can only span a single base-pair, a monointercalation complex forms (Figure 2d). In fact, with bisintercalators the situation is considerably more complicated with the apparent width of the intercalator determining whether the nearest neighbor exclusion principle is obeyed. Although the principle remains poorly understood even today, my idea as a graduate student was to make a bisintercalator that was so rigid it could not form the mono-intercalated complex in Figure 2d. The ultimate goal was to develop a small molecule ligand that might intercalate at sites that lack a conventional neighboring intercalation site, for example, the ends of DNA double helices, replication forks, or abasic sites.

**Figure 3:**
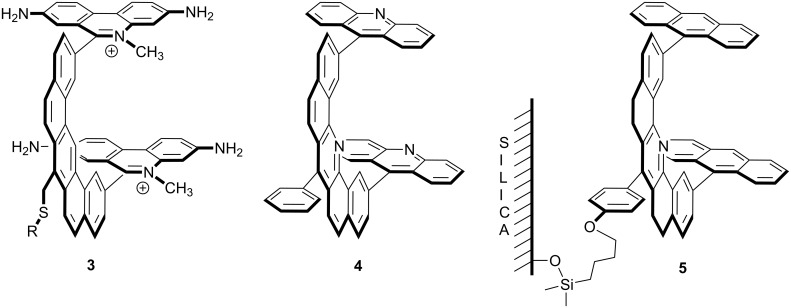
Bismethidium molecular tweezer **3** proposed as a graduate student at Columbia University, which was quite close in structure to **4**, synthesized and studied at Illinois. Chemically bonded stationary phase **5** used for HPLC assay of nitrated aromatics and for quantitative enthalpy determinations.

My original research proposal was submitted August 16, 1983 and was entitled “Synthesis of a Rigid ‘Molecular Tweezer’ with Novel DNA Binding Potential.” The name “molecular tweezer” was inspired by Howard Whitlock’s 1978 report [8] of compound **2** containing two caffeine units linked by a rigid diyne spacer (Figure 2e). Whitlock noted that conventional bisintercalators such as **1** would have their affinity for oligonucleotides significantly reduced as a result of intramolecular π–π aromatic stacking. Whereas the diyne spacer would prevent such stacking, it would not prevent mono-intercalation; therefore, we sought a spacer that would enforce a C-shape on the intercalator units. Compound **3** was one of two highly rigid tweezers proposed (Figure 3). The R-substituent, –CH_2_CH_2_NMe_3_^+^, along with the phenanthridinium units (“wide” chromophores obeying the NEP), were an obvious attempt to provide water solubility to a highly aromatic structure. In Roger Adams’ Laboratory, Craig Vanzyl, Greg Hamilton, and I were able to prepare molecular tweezer **4** and several substituted analogs, but none that were water soluble [9–10].

Fortunately, the excitement surrounding the 1987 Nobel Prize to Cram, Lehn, and Pedersen had generated an enormous interest in host–guest chemistry and there was at this time a move to go beyond cyclic crown ethers. In particular, the groups of Rebek [11] and Hamilton [12] and many others were developing hosts capable of complexing more structurally challenging organic guests such as nucleobases. We quickly discovered that in chloroform solution, **4** and its analogs could bind nitrated aromatic compounds, such as 2,4,7-trinitrofluorenone. Nitrated polycyclic aromatics and polynitrated fluorenones were known pollutants so Kurt Saionz covalently linked the molecular tweezers to silica gel (see **5**, Figure 3), making chemically bonded stationary phases which were packed into HPLC columns that selectively retained and separated nitrated aromatics [13]. The HPLC columns proved to be very useful for quickly measuring Δ*Η*° of complexation and, indeed, multiple guests could be measured at once [14]. The HPLC method of determining complexation Δ*Η*° values was extended by Vincent Kwan to hydrogen bonding host–guest complexes [15].

### Molecular tweezers that complex adenine and analysis of binding interactions

**Figure 4:**
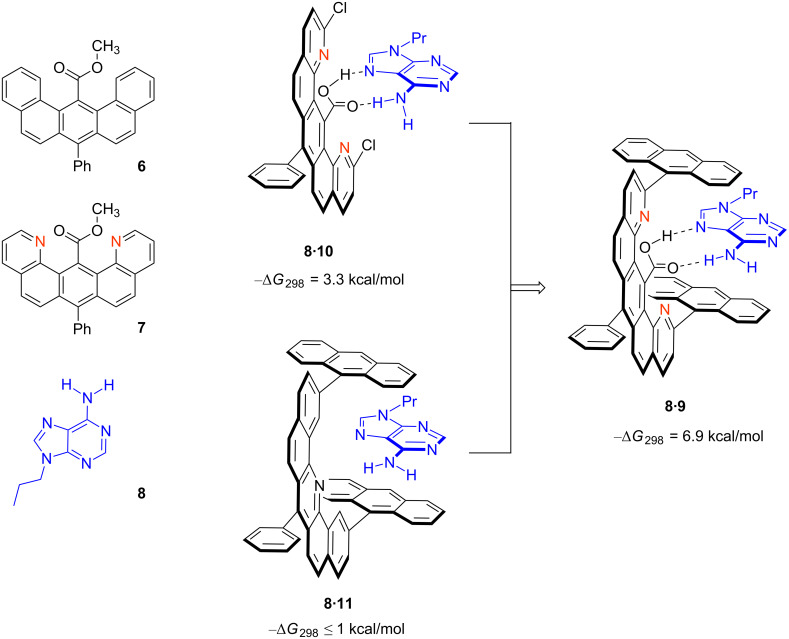
Adenine **8** recognition by carboxylic acid containing tweezer **9** and a component analysis showing “complex additivity” of binding energies.

The idea of incorporating hydrogen bonding functionality into the molecular tweezer was appealing because it meant that aromatic stacking and hydrogen bonding might cooperate to give higher binding constants and guest selectivity. However, the preparation of a rigid aromatic spacer with a functional group converging on the binding cleft was not only a significant synthetic challenge, I questioned whether the group might not distort the spacer, thereby altering its dimensions. I was able to prepare small quantities (<100 mg) of **6** and **7** and crystallize both for X-ray analysis (Figure 4) [16]. The solid state structure of **6** revealed significant distortion and, indeed, a highly nonplanar aromatic spacer that was not suitable for the desired molecular tweezer. In contrast, analog **7** with nitrogen atoms in the peri-positions was a much more planar and suitable candidate.

Weiming Wu was able to prepare molecular tweezer **9** and showed it to bind 9-propyladenine in chloroform with a very high association constant of *K*_assoc_ = 120,000 M^−1^ [17–19]. What role do the hydrogen bonding and aromatic stacking play? As seen in Figure 4, this system provides an excellent example of what Jencks called “complex additivity of binding energies” [20]. The aromatic cleft of molecular tweezer **11** showed no affinity for adenine **8** and the carboxylic acid **10** bound **8** rather weakly (−Δ*G*° = 3.3 kcal/mol), yet the cleft and acid group cooperate together to give the very stable complex **8·9**. One simple interpretation in line with Jencks’ idea is that the entropy paid by the hydrogen bonding is sufficient to allow the enthalpy of the aromatic stacking to be observed. However, there are other explanations for the high stability observed in the **8·9** complex. The aromatic cleft may serve to desolvate the carboxylic acid, thereby improving its hydrogen bonding capability. However, Monte Carlo simulations by Blake and Jorgensen indicate that the cleft of **9** is actually a good host for chloroform and that the carboxylic acid is solvated by more than one chloroform molecule [21]. Another possibility is that the aromatic cleft might somehow decrease the acidity of the acid group making it a better hydrogen bond donor. However, titrations in a mixed aqueous–organic solvent suggest that the carboxylic acid within the molecular tweezer is actually less acidic and likely a less effective hydrogen bonding unit [22].

### Preorganization and cost of freezing single bond rotations

**Figure 5:**
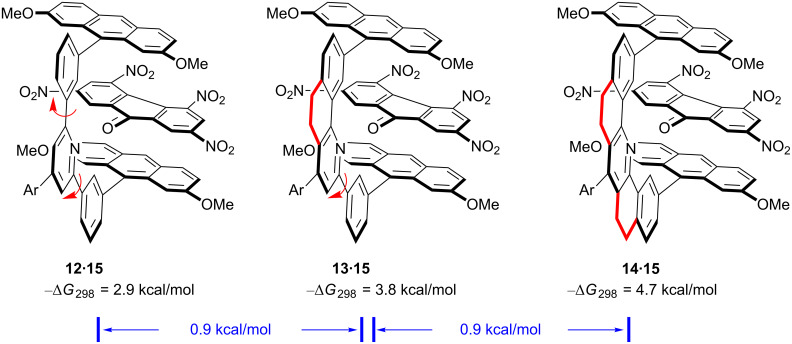
The first determination of the cost of freezing single bond rotations in a host–guest complex.

The studies above show the utility of the molecular tweezer approach in complexing large organic guests while at the same time uncovering important design criteria in host–guest chemistry. Regarding design criteria, the ability to synthesize structurally analogous molecular tweezers provides an unprecedented opportunity to develop a wide range of important structure–property relationships. For example, a lot had been written about the importance of Cram’s preorganization principle in host design, but no one had measured the energy cost of locking a single bond rotation in a host–guest complex. Monica Baloga, Milan Mrksich, and I prepared three new molecular tweezers **12**–**14**, where the spacer units possess zero, one, and two aryl–aryl single bonds (i.e., **14** → **13** → **12**) [23]. Their *K*_assoc_ values with 2,4,5,7-tetranitrofluorenone (**15**) in chloroform were then measured. As seen in Figure 5, freezing each single bond rotation increases complex stability by about 0.9 kcal/mol. This value is in line with a value of *T*Δ*S*° = 0.6 to 1.2 kcal/mol previously suggested by Jencks and Page as the cost paid to freeze out each single bond rotation in a ring-forming reaction [24]. Later Dudley J. Williams, in the context of analyzing the vancomycin complex with D–Ala–D–Ala containing peptides, suggested the cost of freezing a free rotation to be between 0.4 to 0.9 kcal/mol – a value that is also close to what we had measured [25]. In Williams’ case, the value was derived from the entropy of fusion within a homologous series of alkanes, not an analysis of a host–guest system. All these values suggest that freezing out a single bond rotation is not terribly costly but association constants can drop significantly if too much flexibility exists; freezing five single bonds within a complex would lower its stability by as much as 10^4^-fold.

### Research on molecular tweezers goes mainstream

**Figure 6:**
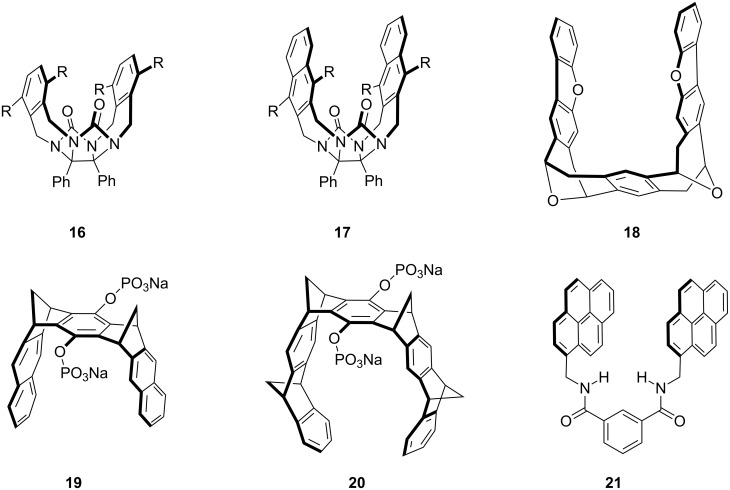
Glycouril-based molecular clip **16** and **17** developed by Nolte, an example of Harmata’s chiral Kagan-ether tweezer **18**, two of Klärner’s water-soluble tweezers (**19** and **20**), and pyrene tweezer **21** studied by Colquhoun.

Since our early studies on molecular tweezers, numerous examples have appeared in the literature. A comprehensive review is not possible, but a few examples from other investigators are presented to illustrate the breadth of structure and function that has been achieved over the past three decades. One of the earliest examples is a molecular tweezer developed contemporaneously with our own efforts. Roeland Nolte and his team first reported the synthesis and X-ray structure of **16** (Figure 6, R = OH) as the basic building block [26]. The now familiar glycoluril motif of **16** clearly has the rigid C-shaped required, and a subsequent report showed it to be capable of complexing dihydroxybenzenes using a combination of aromatic stacking and hydrogen bonding to the urea carbonyl groups [27]. The aromatic stacking surface can be expanded as in molecular tweezer (clip) **17** (Figure 6), but interestingly, this compound is not active whereas the 1,8-substituted naphthalene is, providing a clip that works by an induced fit mechanism (structure not shown) [28]. The Nolte group went well beyond the simple molecular clip architecture with a wide range of molecular and supramolecular architectures derived from the simple glycoluril motif [28].

Another early molecular tweezer developed by Harmata was notable because of its chirality. Harmata and his undergraduate student, Tom Murray (who later obtained his Ph.D. in my group), showed how two units of Kagan’s ether provide the necessary C-shaped geometry [29]. Then, Harmata himself synthesized tweezer **18**, reporting its solution binding studies and a beautiful solid-state inclusion complex with trinitrobenzene [30]. This series of molecular tweezers was reviewed in 2004 [31].

A related class of achiral molecular tweezers was developed by Klärner et al., with their first report appearing in 1996 [32]. These di-, tri-, and tetramethylene-bridged aromatic systems (e.g., **19** and **20**) were prepared by consecutive Diels–Alder reactions and have been shown to exhibit a remarkably rich host–guest chemistry [33]. Perry Corbin, Steven Dell, and I were pleased to work with Frank Klärner and his group on linking a host analogous to **20** to silica and to study the solvent effects on host–guest complexation chemistry using our HPLC method [34].

Many of the molecular tweezers described above were reported many years ago. Were the water-soluble tweezers we set out to prepare in 1985 ever made? Where does the field stand today? We found that our molecular tweezers are well preorganized for dimerization, limiting their use in water [22], whereas Klärner and colleagues discovered that their tweezers **19** and **20** are water-soluble but tend not to self-associate [33]. This has allowed their use in a range of biomolecular recognition applications, which appear quite promising. For example, tweezer **20**, also called CLR01, has been found to bind the accessible lysine and arginine groups of proteins and thereby: (1) inhibit the toxicity of amyloidogenic proteins in cell culture, (2) modulate Aβ protein oligomerization, and (3) disintegrate preformed Aβ fibrils [35]. CLR01 (**20**) was recently shown capable of antagonizing seminal amyloids involved in HIV infection [36].

Beyond the impressive biomedical application described above, very simple molecular tweezers such as **21** have found use in materials and information storage applications. Howard Colquhoun and coworkers have used **21** to recognize sequence information in polymers [37–38], to form healable, supramolecular nanocomposites [39], and even to form supramolecular inkjet printing inks [40]. It is clear that molecular tweezers have found diverse applications, many of which derive from their cleft-like architecture. For example, the recognition of the polymer by a macrocycle would necessitate the threading of the polymer through the macrocycle and the act of reading the polymer sequence would have to be performed sequentially rather than random access.

### Macrocycles make an appearance: macrocyclic bisintercalators and our early efforts to develop DNA probes

**Figure 7:**
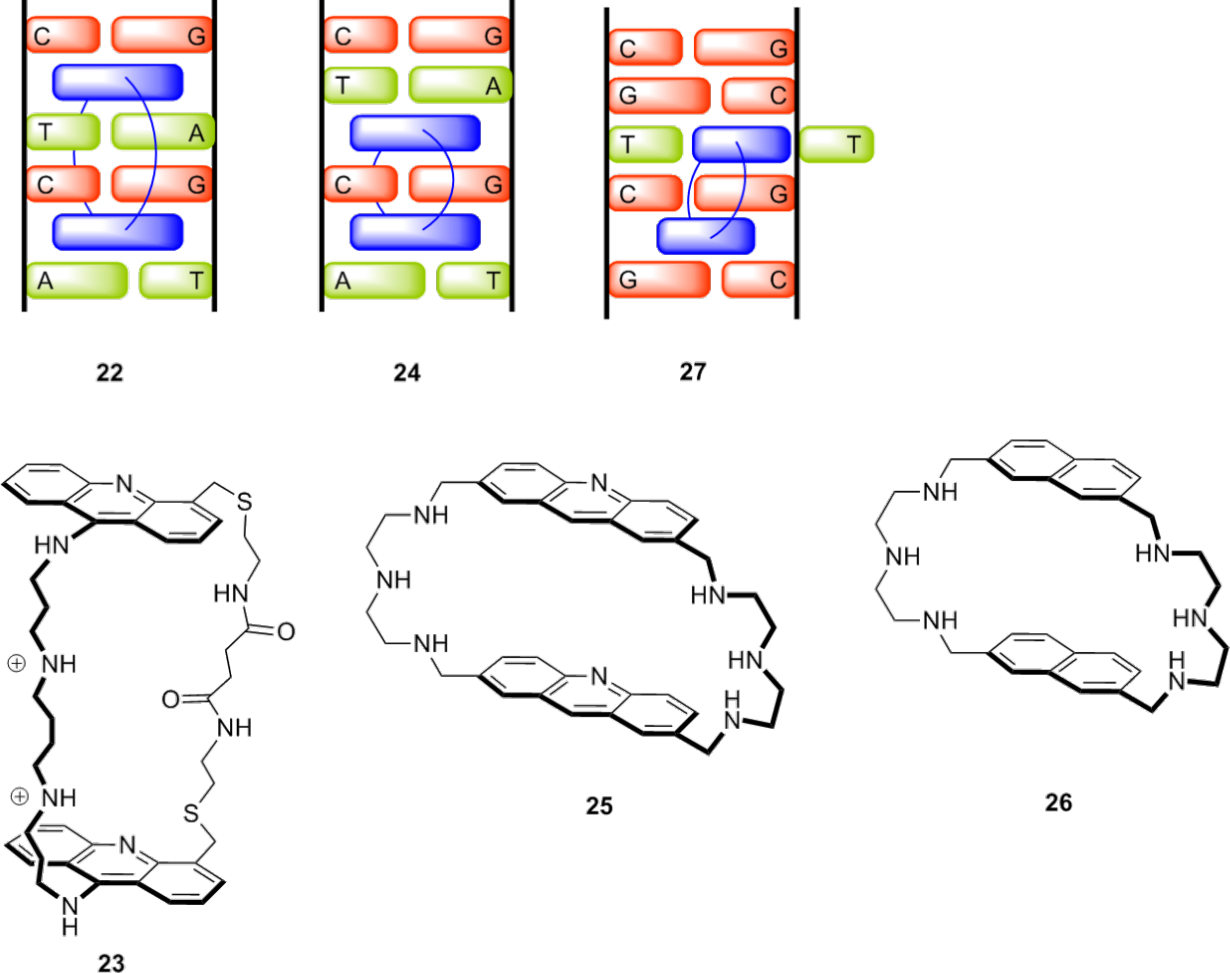
Macrocyclic diacridines and dinaphthalene and the intercalation complexes they may form with one chain in each groove.

As just illustrated, one of the major advantages of the molecular tweezer approach to molecular recognition, and analogously the cleft approach pioneered by Rebek, Hamilton, and others (vide supra), is the ability to recognize large guests, for example, part of the surface of a large macromolecule or biomolecule. Indeed, our original goal of challenging the NEP was guided by the observation that all synthetic bisintercalators of DNA were nonmacrocyclic. DNA bisintercalating natural products with macrocyclic peptide scaffolds (such as echinomycin) were known as early as 1957, but these bound with the macrocycle in a single groove [41]. We wondered whether a macrocyclic bisintercalator that required a linking chain to reside in each groove would be able to bind given that a local melting would be required for one linker to pass to the other side of the double helix. Shown schematically as **22** (Figure 7), such a complex would be an example of what later would be known as a reversible, supramolecular catenane [42–43].

Carol Lamberson synthesized macrocyclic diacridine **23** with spermine and diamide linker chains long enough to allow binding according to the nearest neighbor exclusion principle (e.g., a complex such as **22**). All the data collected showed that this “topologically constrained” bisintercalator was indeed able to insert both acridine rings into double helical DNA; however, we were only able to infer the formation of complex **22** [44]. The data from a combined kinetic, NMR, and modeling study in collaboration with David Wilson were most consistent with complex **24**, although **22** was also consistent with much of the data [45].

Beyond the novelty of the binding mode, we thought these macrocyclic bisintercalators might serve as probes of DNA breathing and further might have particularly slow dissociation kinetics, thereby improving their ability to inhibit enzymatic DNA processing. However, students were not so interested in this area of research, perhaps because I had little training in DNA/small molecule recognition. Whatever the reason, we temporarily suspended this research effort. Fortunately, others were more persevering and pushed the concept forward in important new directions. Earlier work by Jean-Marie Lehn and coworkers showed that bisacridine **25**, which they called cyclobisintercalators, selectively bound DNA hairpins [46]. The same compound was found to selectively photo-cleave abasic sites in DNA [47]. Teulade-Fichou et al. reported that **25** (and especially the naphthalene analog **26**) showed high selectivity in binding pyrimidine mismatches [48]. The affinity of **26** for a TT mismatch in DNA was sufficient to inhibit the binding M. TaqI and, thus, potentially interfere with mismatch repair enzymes that have been implicated in certain diseases [49]. In 2009, Brent Iverson and coworkers reported that a cyclic bisnaphthalenediimide formed a complex with d(CGGTACCG)_2_ that provided a well-resolved NMR and clear NOE signals between the linking chains and both the major and minor grooves [50]. Additional studies on this class of DNA ligands as part of an excellent review of other small molecules recognizing DNA mismatches has been recently published [51].

### Sometimes a break can be productive and refreshing

In the year or so prior to my tenure and promotion, I thought about taking on a new and seemingly more challenging problem. At that time, there was a lot of discussion and some reports on molecular-recognition-driven self-assembly. The grand challenge was to design small subunits that would spontaneously form complex structures noncovalently, ultimately with some particular function. So we began in earnest to develop such systems, focusing primarily on hydrogen bonding. This effort amounted to a nearly 20-year break from DNA intercalators and molecular tweezers. Ed Fenlon, Tom Murray, and Perry Corbin started investigating DNA base-pair analogs and were later joined by Zhanting Li [52], Taiho Park, Jordan Quinn, and Eric Todd [53–56]. The goal was to understand the design principles that lead to high affinity [50,57–58], and then to apply that knowledge to self-assembling systems. Inspired by the work of Lehn [59], Whitesides [60], and Wuest [61], Brook Duerr, Yuguo Ma, Dave Reichert, and Fanwen Zeng developed discrete cyclic assemblies of small molecules [62–63] and dendrimers [64–65] – work that ultimately led Cyrus Anderson, Darrell Kuykendall, Ying Li, Taiho Park, Kwansima Quansah, and Mauricio Suarez to a broader range of supramolecular polymers, including liquid crystals [66], network blends [67], alternating copolymers [68], reversible adhesives [69], and redox-responsive supramolecular blends [70–71].

### The circle of life or circling back within a career?

One of my colleagues at Illinois, John Katzenellenbogen, advised me early in my career not to entirely give up work in any area in which I gained experience. His sage advice was that in time those skills and knowledge, combined with general progress in chemistry could ultimately be leveraged into new and interesting ideas. So around 2007, Jonathan Arambula in my group returned to the idea of molecular-tweezer-like compounds and their ability to bind DNA. But unlike my early research, we were interested in linkers or spacers that minimally allowed or even forced the two chromophores to stack on one another.

**Figure 8:**
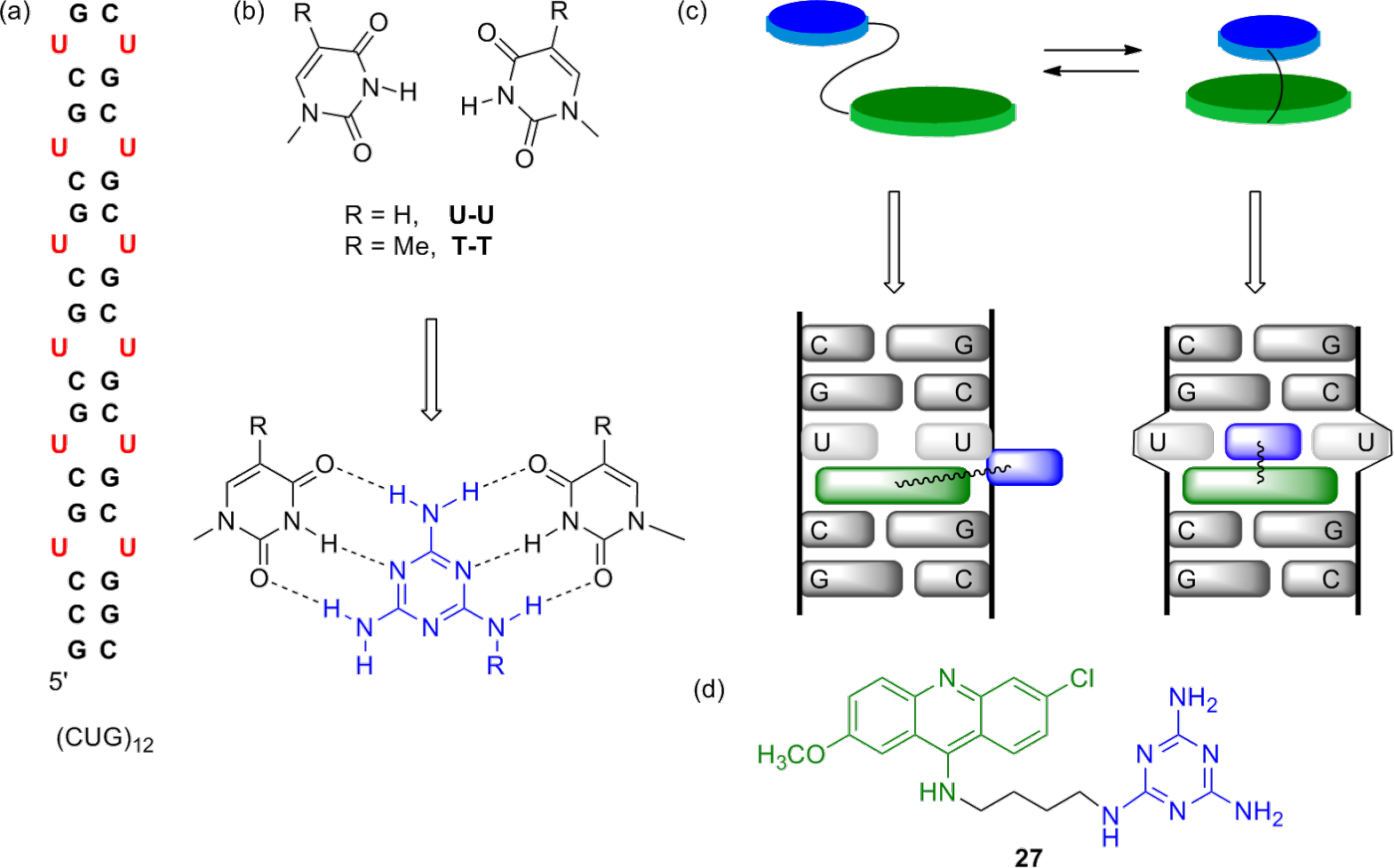
(a) Hairpin structure of CUG^exp^ seen in (CUG)_12_. (b) U–U or T–T mismatches with no inter-strand hydrogen bonds, hypothetically forming base-triplet with 2,4,6-triamino-1,3,5-triazine. (c) Aromatic recognition unit tethered to intercalator in stacked and unstacked conformation. (d) Ligand **27**, an inhibitor of MBNL1N sequestration.

The DNA target in which we became interested was a repeating sequence (CTG)*_n_* in the *DMPK* gene on chromosome 19. The sequence becomes unstable when *n* > 50, undergoing progressive expansion to give CTG^exp^ [72–73]. This inheritable genetic defect, with a frequency of about 1 in 8000 to 1 in 20,000 worldwide, leads to the incurable neuromuscular disease known as myotonic dystrophy type 1 (DM1) [74]. Many of the disease symptoms were attributed to aberrant sequestration of an alternative splicing regulator, MBNL1, by the expanded CUG transcript (CUG^exp^) into nuclear foci [75]. We sought a small molecule that would selectively complex CTG or CUG repeats, both of which were known to form hairpin structures (e.g., see Figure 8a). The structure of r(CUG)_6_, reported by Berglund in 2005 [76], revealed clear opportunities for a rational design approach. The stem-loop structure adopts an overall A-form structure but with little distortion in the backbone that would allow the U–U mismatches to form hydrogen bonds. Our early experience with Janus bases in self-assembly [61,63,77] suggested the use of a melamine (2,4,6-triamino-1,3,5-triazine) unit for formation of a base-triplet, as shown in Figure 8b. Additional reports by Lehn [78] and McLaughlin [79] supported this approach and the melamine unit has more recently been used extensively by Bong [80].

So why did we once again become interested in bisintercalators and the question of rigid vs flexible linkers? The triaminotriazine unit was designed to provide selective recognition of U–U or T–T mismatches, but on its own was viewed as unlikely to provide significant binding affinity because the hydrogen bonding simply involves replacing the hydrogen bonds to water. Coupling the triaminotriazine recognition unit to an intercalator provided the hydrophobic driving force for binding. Of course, nonspecific intercalation would be problematic so here we took advantage of the intramolecular stacking between the intercalator and the recognition unit to reduce off-target binding. Thus, as shown schematically in Figure 8c, the “stacked-intercalator” is too thick to insert between base-pairs but could potentially insert at U–U or T–T sites by forming a base-triplet. Lhomme had shown that nucleic bases tethered to 9-aminoacridines by methylene linker chains, as Whitlock had suggested, preferred stacked structures in water [81]. Nonstacked analogs were shown to recognize and even cleave abasic sites [82].

With this rational design in mind, Jonathan Arambula prepared ligand **27** and a number of control compounds and found it to be a dCTG- and rCUG-selective ligand with apparent *K*_D_ values of about 300–400 nM [83]. A very fruitful collaboration with Anne Baranger’s group was initiated and they developed an electrophoretic mobility shift assay (EMSA) using MBNL1N and r(CUG)_12_ and showed that **27** was indeed an inhibitor of MBNL1N sequestration. However, **27** did not appear to enter cells easily and was poorly soluble and cytotoxic.

**Figure 9:**
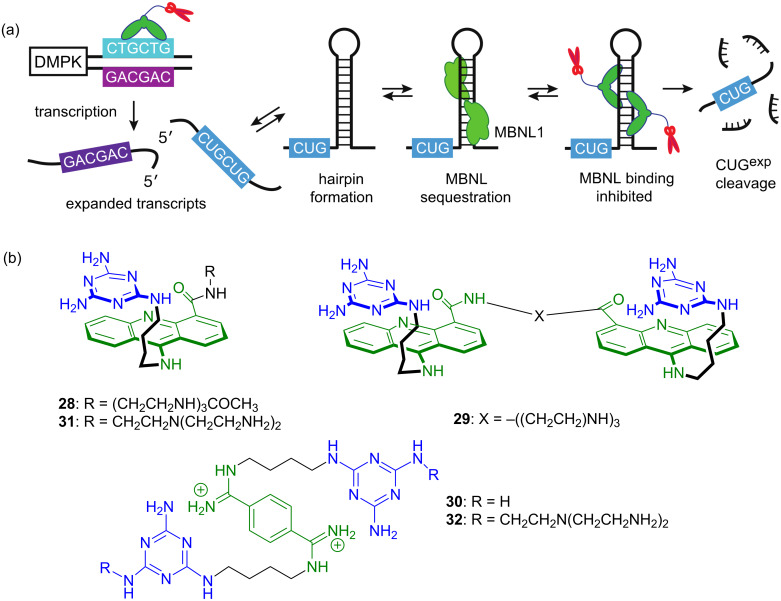
(a) CTG trinucleotide repeat expansion in *DMPK* gene produces expanded transcripts, one which CUG^exp^ sequesters splicing regulator MBNL1. Three modes of disease intervention: binding to CTG^exp^ to inhibit transcription, binding to CUG^exp^ to inhibit MBNL1 binding, and RNase-like cleavage of CUG^exp^. (b) Ligands **28**–**32**.

Amin Jahromi recognized that acridine-containing compounds had been previously induced to enter cells and cell nuclei by attaching amino groups that take advantage of a polyamine transporting system [84–85]. Thus, compound **28** (Figure 9b) was prepared and found to inhibit formation of the MBNL-CUG^exp^ nuclear foci in DM1 model cells. Indeed, it was possible to follow live cells and watch the foci dissolve in real time using time-lapse confocal microscopy [77]. The logical next step was increasing affinity for CUG^exp^ by dimerizing ligands **27**/**28**. Indeed, dimer **29** exhibited a bivalent effect of 133 and was an extremely potent inhibitor of the MBNL1N-(CUG)_12_ complex [86–87].

The possibility of off-target activity by the acridine ligands resulting from the unstacked conformation (Figure 8c) led Chun-Ho Wong to search for an alternative scaffold to drive the recognition [88]. He was attracted to the bisamidinium ion in **30** for three reasons: (1) it is a nuclear localizing agent analogous to nuclear fluorescent stains like DAPI, (2) Butcher reported the NMR structure of DB213, an analogous bisamidinium ligand, bound in the groove of the HIV-1 frameshift site (FS), and (3) the (CUG)_6_ X-ray and HIV-1 FS NMR structures were both A-form and similar suggesting replacement of the ammonium ions in DB213 with triaminotriazine units. Ligand **30** was studied in collaboration with both Anne Baranger and Paul Hergenrother and it was found to have low cytotoxicity, enter DM1 model cells, dissolved the MBNL1 foci and partially corrected the missplicing of two key pre-mRNAs, *cTNT* and *IR*. A terrific collaboration with Professor Edwin Chan’s group at the Chinese University of Hong Kong allowed the compounds to be tested in vivo using a DM1 *Drosophila* model that looked at the rough eye phenotype with i(CUG)_480_ flies. Ligand **30** showed significant and dose-dependent improvement in the rough eye phenotype, whereas the negative control, DB213 showed much weaker activity. Much less effort has been devoted to developing small molecules to treat DM2, which originates in a CCUG expansion, but Lien Nguyen, Chun-Ho Wong, and JuYeon Lee used similar rational design approaches and found lead agents that are selective for this RNA as well [89].

Although the gain of function mechanism that has CUG^exp^ sequestering MBNL1 is well supported, it is clear that the disease pathobiology is more complex. For example, Ranum recently reported [90] that both the CUG^exp^ and CAG^exp^ undergo repeat-associated non-ATG (RAN) translation to produce up to nine homopeptides, some of which are known to be toxic and involved in other disease [91]. Lien Nguyen and Long Luu considered the possibility of rationally designing ligands that could operate on multiple targets in the DM1 pathobiology. They designed and studied ligands such as **31** and **32**. These ligands were shown to bind the DNA that causes DM1, interacting with CTG^exp^ to inhibit transcription to CUG^exp^, also binding CUG^exp^ that slips through inhibiting its sequestration of MBNL1, and, with the catalytic amino/ammonium/imidazole groups, slowly cleaving the CUG-RNA to prevent RAN translation [92]. Edwin Chan and his group again studied the in vivo activity of our compounds, showing that **32** reversed two separate CUG^exp^-induced phenotypes in transgenic DM1 *Drosophila*, specifically the rough eye phenotype and larvae crawling mobility.

## Conclusion

### Detours, perspectives, and future studies

I was Department Head or Interim Department Head for a total of eight years (1999–2000 and 2005–2012). It was an honor to serve our outstanding faculty, staff, and students and to follow luminaries such as William A. Noyes, Roger Adams, Herb Carter, Larry Faulkner, and Gary Schuster. Being department head was without a doubt the most difficult thing I did during my career. It was a period of extraordinary personal growth, having learned how to work with a wide range of people and manage a complex organization. The expressions “herding cats” and “drinking from a fire hose” are apropos descriptors as it was more than a full time job and extremely demanding in other ways. Although it was hard to take time away from research and teaching, the department head job was interesting, challenging, and highly rewarding in seeing the department move forward, especially with the help of our loyal alumni.

We entitled the thematic issue containing this contribution “Supramolecular chemistry at the interface of biology, materials and medicine.” Some of the examples presented herein illustrate the potential of the supramolecular approach to lead to advanced therapeutic agents. In particular the Klärner molecular tweezers that complex lysine-containing peptides may lead to agents that dissolve Alzheimers plaques or inhibit their formation. Our own efforts to create small molecules to target the toxic RNA involved in myotonic dystrophy have expanded to include multitarget drug-discovery approaches where supramolecular design principles led to DNA and RNA-selective small molecules that function even in the complex organisms (i.e., *Drosophila*).

Do we know so much about supramolecular interactions that all of the focus now should be on applications in biology, materials and medicine? The answer to this question is emphatically “No!” At a recent NSF workshop comprised of physical chemists and supramolecular chemists focused on water, the supramolecular chemists mostly agreed that water was not a special solvent, it just occupied an extreme, with low polarizability and a high cohesive nature. In stark contrast, the physical chemists showed plots indicating that water was unlike any other liquid and was clearly special. There also remain debates about whether the π-cation or face-edge aromatic interactions are unusual or even important. Therefore, model studies that shed light on the strength and nature of supramolecular contacts continue to be critically important.

One important emerging area I would like to highlight is the development of complex supramolecular systems. So much of supramolecular chemistry is inspired by biology, it is only natural that the complexity of biological systems be modeled in supramolecular systems. Thus, future developments will lead to multicomponent supramolecular structures/systems that evolve over time or communicate or respond to external or internal stimuli. Dynamic covalent chemistry has moved in this direction [93], and some initial efforts in this area using supramolecular chemistry have also appeared. For example, Andy Wilson’s group reported a sequence of supramolecular recognition events that proceed in a controlled and defined manner, the specific pathway guided by what is present in solution [94]. This primitive model of a signaling cascade points to what may be possible as this area develops.
